# Tumor necrosis factor alpha neutralization attenuates immune checkpoint inhibitor-induced activation of intermediate monocytes in synovial fluid mononuclear cells from patients with inflammatory arthritis

**DOI:** 10.1186/s13075-022-02737-6

**Published:** 2022-02-14

**Authors:** Anne Sofie Sørensen, Morten Nørgaard Andersen, Kristian Juul-Madsen, Amalie Dyrelund Broksø, Cæcilie Skejø, Henrik Schmidt, Thomas Vorup-Jensen, Tue Wenzel Kragstrup

**Affiliations:** 1 Department of Biomedicine, Aarhus University, Skou Building, DK-8000 Aarhus C, Denmark; 2 Department of Clinical Biochemistry, Aarhus University Hospital, Aarhus N, Denmark; 3 Department of Oncology, Aarhus University Hospital, Aarhus N, Denmark; 4 Department of Rheumatology, Aarhus University Hospital, Aarhus N, Denmark; 5 Diagnostic Center, Silkeborg Regional Hospital, Silkeborg, Denmark

**Keywords:** Immunotherapy, Arthritis, Cytokine, Monocyte

## Abstract

**Objective:**

During treatment with immune checkpoint inhibitors (ICI) such as the anti-PD-1 antibody pembrolizumab, half of patients with pre-existing inflammatory arthritis experience disease flares. The underlying immunological mechanisms have not been characterized. Here, we investigate the effect of pembrolizumab on cells involved in inflammation and destruction in the synovial joint and how immunosuppressive treatments affect the pembrolizumab-induced immune reactions.

**Methods:**

We included synovial fluid mononuclear cells (SFMCs, *n* = 28) and peripheral blood mononuclear cells (PBMCs, *n* = 6) from patients with rheumatoid arthritis and peripheral spondyloarthritis and PBMCs from healthy controls (*n* = 6). Fibroblast-like synovial cells (FLSs) were grown from SFMCs. The in vitro effect of pembrolizumab was tested in SFMCs cultured for 48 h, FLS-PBMC co-cultures and in SFMCs cultured for 21 days (inflammatory osteoclastogenesis). Cells and supernatants were analyzed by ELISA, flow cytometry, and pro-inflammatory multiplex assay. Finally, the effect of the disease-modifying anti-rheumatic drugs (DMARDs) adalimumab (TNFα inhibitor), tocilizumab (IL-6R inhibitor), tofacitinib (JAK1/JAK3 inhibitor), and baricitinib (JAK1/JAK2 inhibitor) on pembrolizumab-induced immune reactions was tested.

**Results:**

Pembrolizumab significantly increased monocyte chemoattractant protein-1 (MCP-1) production by arthritis SFMCs (*P* = 0.0031) but not by PBMCs from patients or healthy controls (*P* = 0.77 and *P* = 0.43). Pembrolizumab did not alter MMP-3 production in FLS-PBMC co-cultures (*P* = 0.76) or TRAP secretion in the inflammatory osteoclastogenesis model (*P* = 0.28). In SFMCs, pembrolizumab further increased the production of TNFα (*P* = 0.0110), IFNγ (*P* = 0.0125), IL-12p70 (*P* = 0.0014), IL-10 (*P* = 0.0100), IL-13 (*P* = 0.0044), IL-2 (*P* = 0.0066), and IL-4 (*P* = 0.0008) but did not change the production of IL-6 (*P* = 0.1938) and IL-1 (*P* = 0.1022). The SFMCs treated with pembrolizumab showed an increased frequency of intermediate monocytes (*P* = 0.044), and the MCP-1 production increased only within the intermediate monocyte subset (*P* = 0.028). Lastly, adalimumab, baricitinib, and tofacitinib treatment were able to attenuate the pembrolizumab-induced MCP-1 production (*P* = 0.0004, *P* = 0.033, and *P* = 0.025, respectively), while this was not seen with tocilizumab treatment (*P* = 0.75).

**Conclusion:**

Pembrolizumab specifically activated intermediate monocytes and induced the production of several cytokines including TNFα but not IL-6. These findings indicate that flares in patients with pre-existing inflammatory arthritis involve monocyte activation and could be managed with TNFα neutralization.

**Supplementary Information:**

The online version contains supplementary material available at 10.1186/s13075-022-02737-6.

## Introduction

During cancer treatment with immune checkpoint inhibitors (ICI), half of patients with pre-existing inflammatory arthritis have disease flares [1]. Such reactions are part of the immune activation often seen with ICI treatment and are termed immune related adverse events (IRAEs). These disease flares resemble disease activity usually seen in patients with inflammatory arthritis including swollen and tender joints. However, the specific underlying mechanisms of ICI-associated inflammatory arthritis have not been studied.

The programmed death 1 (PD-1) receptor and its ligands PD-L1 and PD-L2 are co-inhibitory receptors, which negatively regulate T cell activity, for this reason usually referred to as immune checkpoints [2–5]. The recently developed ICIs function by blocking these co-inhibitory receptors, e.g., pembrolizumab is a blocking antibody targeting the PD-1 receptor. By removing the inhibitory signals in the immune system, a now well-described series of events induce anti-cancer effects mediated primarily by CD8+ T cells [3, 6, 7]. However, it is not known whether IRAEs are a result of stimulated CD8+ T cells or the consequence of activation of other parts of the immune system.

The pathogenesis of traditional immune-mediated inflammatory arthritis encompasses both systemic inflammation and a more local inflammatory response promoting joint destruction [8]. Cells of the mononuclear phagocytic system, especially monocytes and macrophages, are major factors in the inflammatory process. Monocytes are divided into three subsets, namely the classical monocytes (CD14+CD16−), intermediate monocytes (CD14+CD16+), and non-classical monocytes (CD14−CD16+) [9, 10]. While the classical monocytes are the most abundant type in healthy individuals, the CD16^+^ monocytes increase during inflammation, including in autoimmune diseases [10, 11]. When activated, they produce pro-inflammatory cytokines including tumor necrosis factor alpha (TNFα), IL-6, and monocyte chemoattractant protein 1 (MCP-1) [12, 13]. The joint destruction in inflammatory arthritis is primarily mediated by fibroblast-like synoviocytes (FLS) and osteoclasts. These cells produce matrix metalloproteinases (e.g., matrix metalloproteinase-3, MMP-3) and enzymes (e.g., tartrate-resistant acid phosphatase, TRAP), which facilitate cartilage breakdown and bone destruction [14, 15].

Here, we investigate the effect of pembrolizumab on cells involved in inflammation and destruction in the synovial joint and how immunosuppressive treatments affect the pembrolizumab-induced immune reactions. We specifically hypothesized that monocytes are activated in pembrolizumab-induced immune reactions seen in patients with pre-existing inflammatory arthritis.

## Materials and methods

### Study population and ethics

The study population (Table 1) consists of synovial fluid mononuclear cells (SFMCs, *n* = 28) from patients with RA (*n* = 14), peripheral spondyloarthritis (SpA) (*n* = 9), and PsA (*n* = 5). Additionally, peripheral blood mononuclear cells (PBMCs) from 6 of these patients (3 SpA and 3 RA patients) were obtained. None of these patients had a cancer diagnosis or had been treated with ICI therapy in vivo. There were two main inclusion criteria in this study. (1) The patients had to fulfill either the EULAR/ACR classification criteria for RA, the ASAS classification criteria for peripheral SpA, or the CASPAR criteria for PsA [16–18]. (2) We also included patients with at least one swollen joint requiring joint fluid aspiration as part of therapy. The synovial fluid was primarily obtained from large joints such as knee, shoulder, and ankle joints. The exact anatomical site was not registered as part of sample collection. PBMCs (*n* = 6) from healthy controls were obtained from the blood bank (Department of Immunology, Aarhus University Hospital). The number of patients in each experiment is listed in the figure legends.

**Table 1:** Patient characteristics

Patient characteristics			
Diagnosis	RA (***n*** = 14)	Peripheral SpA (***n*** = 9)	PsA (***n*** = 5)
Age (years)	53 (36.5– 59)	40 (33–43)	45 (35–49)
Gender (females)	5	4	3
**Disease activity**			
CRP (mg/L)	19 (8.7–20.75)	16.25 (5.5–44.13)	4 (3–5)
DAS28CRP	3.71 (3.19–4.12)	3.50 (2.90–4.66)	3.01 (2.79–3.68)
Swollen joint count	1 (1–2.75)	1.5 (1–2)	1.5 (0.75–5.25)
Tender joint count	1 (1–2.5)	1 (1–2)	1.5 (0.75–3)
Disease duration (years)	11 (5–18)	2 (0.5–15)	14 (5–14)
RF positive (*n*)	4	0	0
Anti-CCP positive (*n*)	4	0	0
HLA-B27 (*n*)	–	5	–
**Treatment**			
**csDMARDs**			
MTX (*n*)	10	2	3
Salazopyrine (*n*)	3	3	0
**bDMARDs**			
TNF-inhibitor (*n*)	4	4	1
**No DMARDs (*n*)**	2	4	2

Data are expressed as median and IQR

Missing data (*n*): age (1), gender (1), CRP (1), patient global VAS (6), swollen joint count (1), tender joint count (1), disease duration (1), RF (1), anti-CCP (1), treatment (1)

*Abbreviations*: *RA* rheumatoid arthritis, *SpA* spondyloarthritis, *PsA* psoriatic arthritis, *CRP* C-reactive protein, *DAS28CRP* Disease Activity Score 28 based on CRP, *RF* rheumatoid factor, *Anti-CCP* antibodies targeting citrullinated peptides, *csDMARDs* conventional synthetic disease-modifying anti-rheumatic drugs, *bDMARDs* biologic disease-modifying anti-rheumatic drugs, *IQR* interquartile range

### Isolation of mononuclear cells

SFMCs and PBMCs were isolated using Ficoll-Paque density centrifugation. The isolated cells were cryopreserved in freeze medium (70% [v/v] RPMI-1640 + 20% [v/v] heat-inactivated FCS + 10% [v/v] DMSO) and stored at − 135 °C until use.

### The 48-h SFMC and PBMC models

The SFMC and PBMC cultures primarily consisted of monocytes and lymphocytes. The cells were thawed at 37 °C and seeded at a concentration of 2 million cells/mL in DMEM with 10% FCS and penicillin and streptomycin as done previously [19]. SFMCs and PBMCs were cultured with pembrolizumab at 5 μg/mL for 48 h. In all experiments, culture medium and lipopolysaccharide (LPS) at 100 ng/mL (Sigma-Aldrich, St. Louis, MO, USA) were included as negative and positive controls, respectively [20]. After 48 h of incubation, cell suspensions were transferred to Eppendorf tubes and centrifuged at 300 × *g*, 10 min, RT. The supernatants were collected and stored until later analysis by MCP-1 ELISA and the V-plex pro-inflammatory panel as previously described [21].

### Monocyte flow cytometry

SFMCs were seeded at a concentration of 1 million cells/mL. Brefeldin A (Sigma-Aldrich) was added during the last 4 h of incubation at 10 μg/mL. Non-adherent cells were harvested by washing the plates with culture medium. The adherent cells were detached using a detachment buffer (PBS/0.5% BSA/5 mM EDTA/4 mg/mL Lidocaine) for 10 min at 37 °C followed by scraping with a sterile cell scraper. The cells were stained with antibodies to CD45 conjugated with AF700 (clone: HI30, BD bioscience, Albertslund, Denmark), CD16 conjugated to PE-Cy7 (clone: 3G8, Biolegend, San Diego, CA, USA), CD14 conjugated to V500 (Clone: MφP9, BD Bioscience), TLR-2 conjugated to BB700 (clone: 11G7, BD bioscience,), and live/dead viability dye (Life Technologies, Naerum, Denmark). To prevent non-specific staining, the cells were blocked with human IgG at 100 μg/mL (Beriglobin, CSL Behring, King of Prussia, PA) and phosphorothyoate-oligo-deoxynucleotides 10 μg/mL [22, 23]. After surface staining, cells were fixed using 4% (v/v) formaldehyde diluted in PBS and permeabilized using 0.2% (w/v) saponin (Sigma-Aldrich) in PBS with 0.5% (w/v) BSA and 0.09% (v/v) NaN_3_ (permeabilization buffer). Then, intracellular staining was performed with MCP-1 conjugated to PE (clone: 5D3-F7, BD bioscience) and cells were run on a LSR Fortessa flow cytometer (BD Bioscience) [24]. Fluorescence minus one (FMO) controls for MCP-1 and CD16 as well as unstained samples were included to determine the threshold for positive staining. Analysis of data was performed in FlowJo version 10.5.0 for Mac. The gating strategy used to identify monocyte subsets was confirmed by staining PBMCs from healthy controls (Supplementary, figure S1).

### Cell culture treatment with immunosuppressive drugs

SFMCs were seeded in a density of 2 million cells/mL. SFMCs were cultured with pembrolizumab at 5 μg/mL with or without the TNFα inhibitor adalimumab (Humira™, Abbvie, North Chicago, IL, USA) at 5 μg/mL, the IL-6R inhibitor tocilizumab (RoActemra™, Roche, Hvidovre, Denmark) at 5 μg/mL or the Janus kinase (JAK) inhibitors tofacitinib citrate at 200 nM (Selleckchem, Munich, Germany), or baricitinib citrate at 200 nM (Selleckchem) as described previously [25, 26]. In all experiments, negative controls included untreated cells or the vehicle dimethyl sulfoxide (DMSO). Cell cultures treated with LPS at 100 ng/mL were included as positive controls for cytokine expression. After 48 h of incubation, cell suspensions were transferred to Eppendorf tubes and centrifuged at 300 × *g* for 10 min at RT. The supernatants were collected and stored until later analysis for MCP-1 production.

### MCP-1 and MMP3 ELISA and TRAP measurement

The concentration of MCP-1 (Biolegend) and MMP-3 (R&D Systems) were analyzed by commercially available enzyme-linked immunosorbent assays (ELISAs) according to the manufacturer’s instructions. The concentration of TRAP was analyzed by an enzymatic assay (B-bridge International) according to the manufacturer’s instructions.

### V-plex pro-inflammatory panel 1

The supernatants were analyzed for the production of 10 different pro-inflammatory cytokines (IFNγ, IL-1β, IL-2, IL-4, IL-6, IL-8, IL-10, IL-12p70, IL-13, and TNFα) using a V-plex pro-inflammatory panel 1 kit (Meso Scale Discovery, catalog number: K15049D-1, V-plex pro-inflammatory panel 1) according to the manufacturer’s instructions. IL-8 was excluded because all data points were above the detection range.

### Statistics

Statistical analyses and graphs were done using GraphPad Prism 7. Data were normalized into ratios by dividing the value of each sample with the value of the negative control cultures. Whether data followed a normal distribution was assessed by QQ-plots and histograms. Log transformation of ratios was used to achieve a normal distribution and a paired Student’s t-test was performed. Statistical significance was based on a *P*-value < 5%.

## Results

### Pembrolizumab increases MCP-1 production in SFMCs but not in PBMCs

**Fig. 1:**
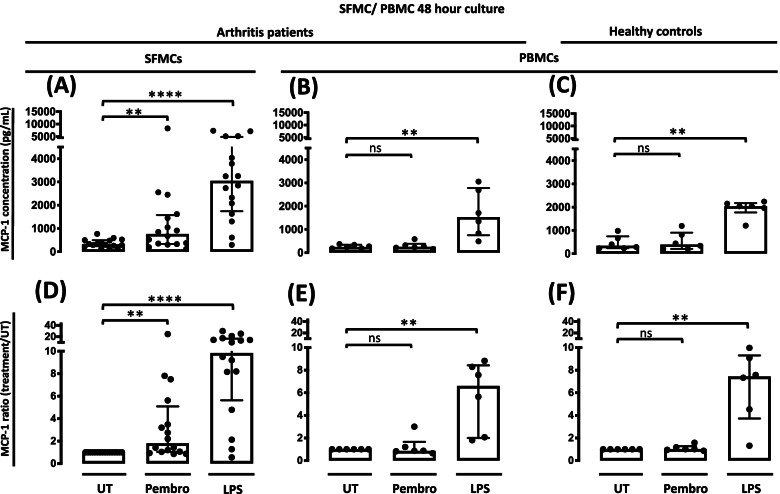
MCP-1 production following treatment with pembrolizumab. **A**–**C** MCP-1 production in SFMCs (*n* = 16, 3 SpA patients, 5 PsA patients, and 8 RA patients) and PBMCs (*n* = 6, 3 SpA patients and 3 RA patients) from patients and healthy controls (*n* = 6) cultured for 48 h untreated (UT) or treated with pembrolizumab (Pembro) or LPS. **D**–**F** Data were normalized to untreated cultures and expressed as ratios. Data is presented as median with interquartile range. Log-transformed ratios were analyzed with the paired Student’s *t*-test. *P-value < 0.05, **P-value < 0.01, ***P-value < 0.001

First, we wanted to study the effects of pembrolizumab in vitro to test the hypothesis that mononuclear cells from an inflamed environment would be more sensitive to pembrolizumab treatment. We compared SFMCs from patients with inflammatory arthritis with PBMCs from both patients and healthy controls. Pembrolizumab only increased the MCP-1 production in the SFMC cultures (*P* = 0.0031), whereas PBMCs from both healthy controls and patients were not affected by the pembrolizumab treatment (*P* = 0.43 and *P* = 0.77, respectively) (Fig. 1). This indicated that the immune checkpoint inhibition caused by pembrolizumab only induced immunological reactions in cells already activated in vivo. In contrast, LPS increased the MCP-1 production in both SFMC and PBMC cultures (SFMCs, *P* < 0.0001; arthritis PBMCs, *P* = 0.0031; HC PBMCs, *P* = 0.0026) (Fig. 1). Taken together, this indicates that arthritis SFMCs can be used to study pembrolizumab-induced immunological reactions. Furthermore, no visual difference was seen when comparing response to pembrolizumab in SFMCs from RA, SpA, and PsA patients (Supplementary, figure S1).

### Pembrolizumab does not increase the production of MMP-3 or TRAP

**Fig. 2:**
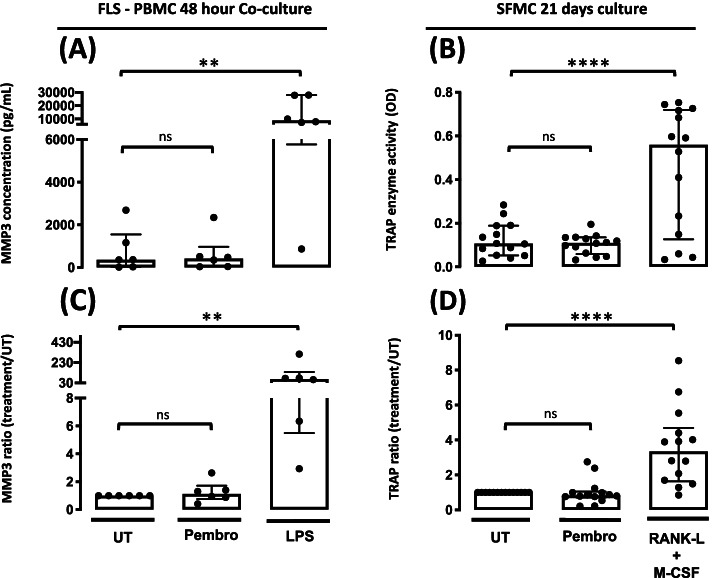
MMP-3 and TRAP production following treatment with pembrolizumab. **A** MMP-3 production in the FLS-PBMC co-culture cultured for 48 h untreated (UT) or treated with pembrolizumab (Pembro) or LPS (*n* = 6, 3 SpA patients and 3 RA patients). **B** TRAP activity in SFMCs cultured for 21 days untreated (UT) or treated with pembrolizumab (Pembro) or LPS (*n* = 14, 7 SpA patients and 7 RA patients). **C**, **D** Data were normalized to untreated cultures and expressed as ratios. Data is presented as median with interquartile range. Log-transformed ratios were analyzed with the paired Student’s *t*-test. *P-value < 0.05, **P-value < 0.01, ***P-value < 0.001, ****P-value < 0.0001

Inflammatory arthritis is often characterized by joint destruction. Therefore, we investigated whether pembrolizumab increased the production of proteases and enzymes involved in this process. When treating the FLS-PBMC co-cultures with pembrolizumab, no increase in MMP-3 production was seen (*P* = 0.76). Similarly, in SFMCs cultured for 21 days with pembrolizumab, no difference in TRAP secretion was observed (*P* = 0.28) (Fig. 2).

### Pembrolizumab increases the frequency of synovial fluid intermediate monocytes

**Fig. 3:**
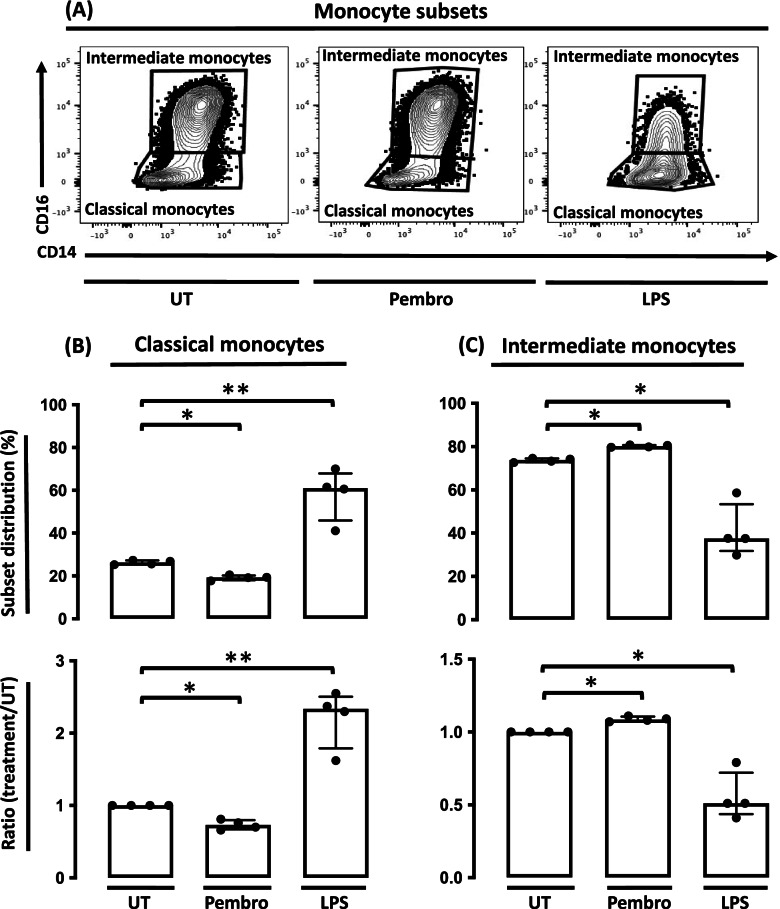
Distribution of the monocyte subsets among SFMCs following treatment with pembrolizumab (*n* = 4, 2 patients with SpA and 2 patients with PsA). **A** Representative CD14 vs. CD16 dot plot of monocyte subsets (CD14+CD16−: classical monocytes; CD14+CD16+: intermediate monocytes) in untreated cultures (UT), pembrolizumab treated cultures (Pembro), and LPS-treated cultures (LPS). **B** Upper panel: Frequency of classical monocytes in each culture. Lower panel: Data were normalized to untreated cultures and expressed as ratios. **C** Upper panel: Frequencies of intermediate monocytes in each culture. Lower panel: Data were normalized to untreated cultures and expressed as ratios. Data is presented as median with interquartile range. Log-transformed ratios were analyzed with the paired Student’s *t*-test. *P-value < 0.05, **P-value < 0.01

Next, we used intracellular flow cytometry to characterize the cell type responsible for the MCP-1 production. We characterized the monocytes as CD45^+^/Live/TLR-2^+^/single cells (Supplementary, figure S2 and S3). These cells were then divided into subsets based on their expression of CD14 and CD16 as done previously [27, 28]. Here, SFMCs treated with pembrolizumab showed a small but consistent increase in the frequency of intermediate monocytes (*P* = 0.044) and a concomitant decrease in the frequency of classical monocytes (*P* = 0.047) compared with untreated cultures. In contrast, cultures treated with LPS showed an increase in the classical monocytes and a decrease in the intermediate monocytes (*P* = 0.0045 and *P* = 0.021, respectively) (Fig. 3).

### Pembrolizumab increases the MCP-1 production in intermediate monocytes but not in classical monocytes

**Fig. 4:**
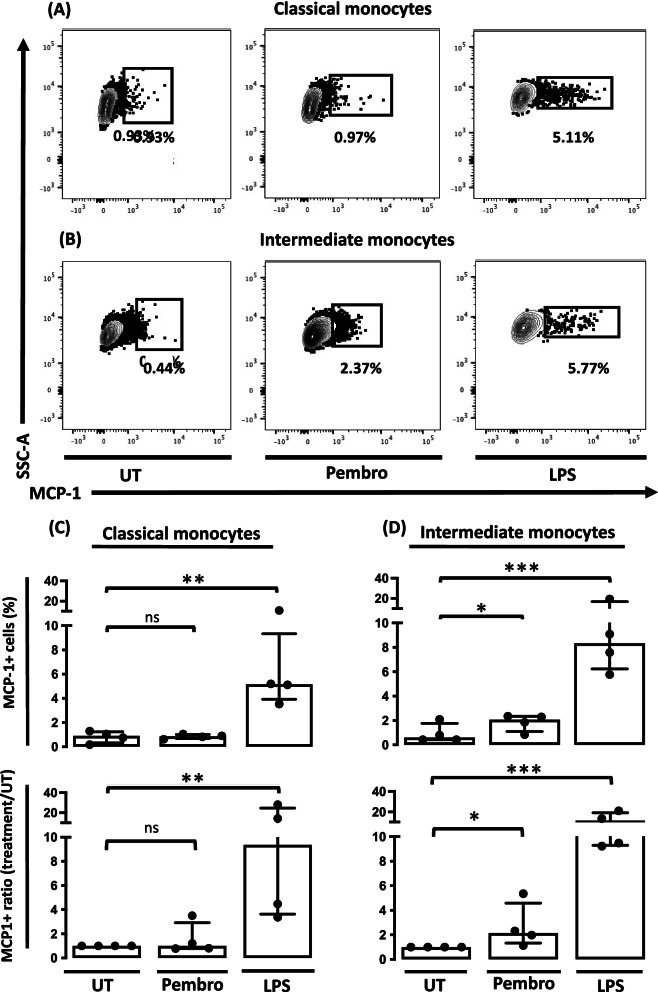
MCP-1 production in the monocyte subsets following treatment with pembrolizumab in SFMCs from arthritis patients (*n* = 4, 2 SpA patients and 2 PsA patients). **A** Representative dotplots of MCP-1 production in classical monocytes. **B** Representative dot plots of MCP-1 production in intermediate monocytes. **C** Upper panel: Frequency of MCP-1+ cells in classical monocytes in each culture. Lower panel: Data were normalized and expressed as ratios. **D** Upper panel: Frequency of MCP-1+ cells in intermediate monocytes in each culture. Lower panel: Data were normalized and expressed as ratios. All data are expressed as median with interquartile range. Log-transformed ratios were analyzed with the paired Student’s *t*-test. *P-value < 0.05, **P-value < 0.01, ***P-value < 0.001. UT, untreated; Pembro, pembrolizumab

Then, MCP-1 production in all monocytes as well as the different monocyte subsets was evaluated using intracellular flow cytometry. Pembrolizumab increased the MCP-1 production when gating on all monocytes (*P* = 0.0191) supporting findings made by ELISA (supplementary, figure S4). Strikingly, however, pembrolizumab increased the MCP-1 production specifically in the intermediate monocytes (*P* = 0.028) but not in the classical monocytes (*P* = 0.32) (Fig. 4). In contrast, LPS increased the production of MCP-1 in both intermediate monocytes (*P* = 0.0010) and classical monocytes (*P* = 0.0221) (Fig. 4).

### Pembrolizumab increased the production of TNF-α, IL-10, IL-12p70, IFN-γ, IL-13, IL-2, and IL-4 but not IL-6 and IL-1

**Fig. 5:**
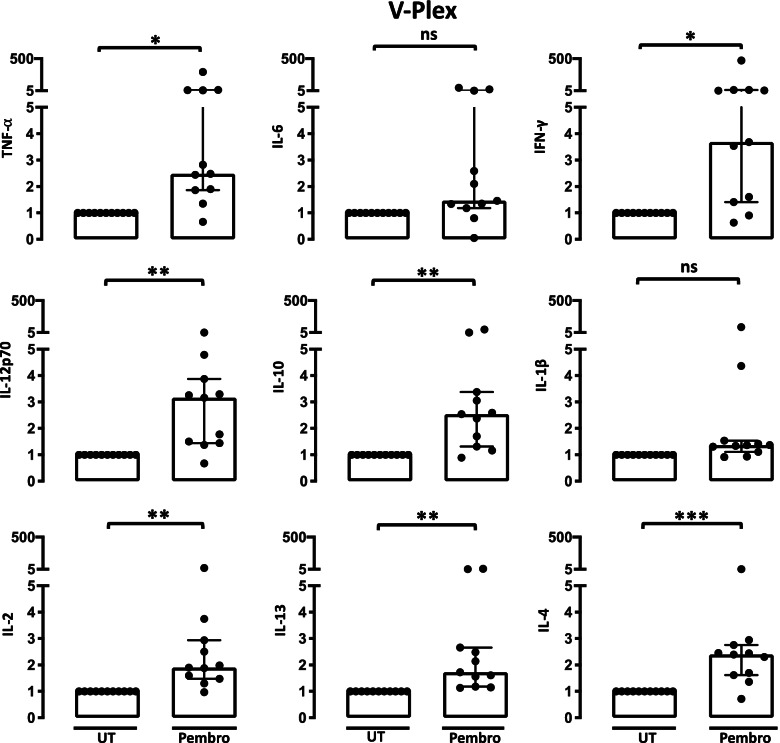
Production of different pro-inflammatory cytokines by SFMC cultures following treatment with pembrolizumab (*n* = 11). Production of TNF, IL-6, IFN-γ, IL-12p70, IL-10, IL-1β, IL-2, IL-13, and IL-4 in SFMCs from arthritis patients cultured for 48 h untreated (UT) or treated with pembrolizumab (Pembro) (8 RA patients, 2 PsA patients, and 1 SpA patient). Data were normalized to untreated cultures and expressed as ratios. Cytokine measurements were excluded if levels were above or below the detection range. All data are expressed as median with interquartile range. Log-transformed ratios were analyzed with the paired Student’s *t*-test. *P-value< 0.05, **P-value < 0.01, ***P-value< 0.001

Now, we investigated the cytokine profile induced by pembrolizumab in more detail with the V-plex pro-inflammatory multiplex panel. Pembrolizumab significantly increased the production of TNFα (*P* = 0.0110), IL-10 (*P* = 0.0100), IL-12p70 (*P* = 0.0014), IL-13 (*P* = 0.0044), IFNγ (*P* = 0.0125), IL-2 (*P* = 0.0066), and IL-4 (*P* = 0.0008). Interestingly, however, IL-6 and IL-1 did not increase in pembrolizumab treated cultures (*P* = 0.1938, *P* = 0.1022) (Fig. 5).

### TNFα and JAK/STAT inhibitors decreased the pembrolizumab-induced MCP-1 production but IL-6R inhibition did not

**Fig. 6:**
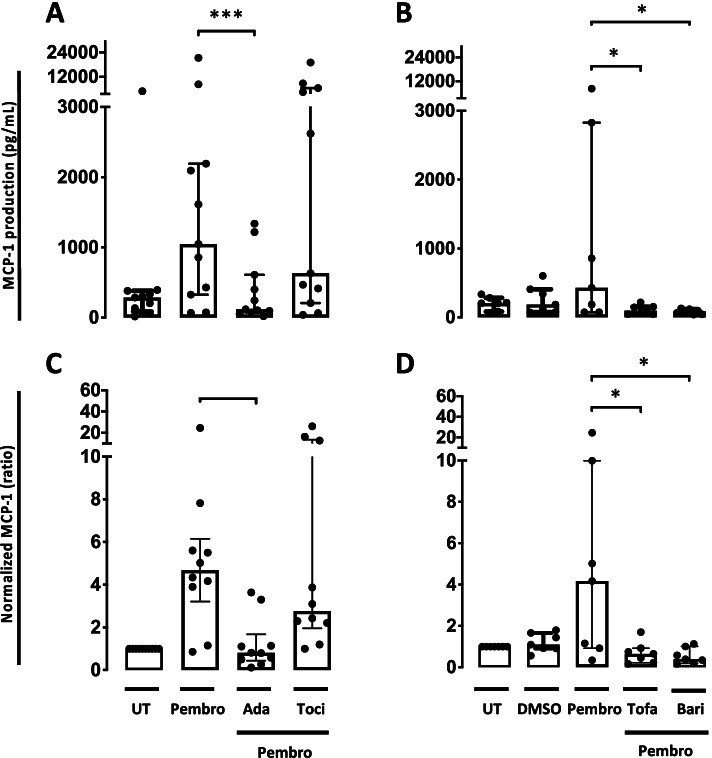
MCP-1 production in SFMC cultures treated with pembrolizumab combined with different DMARDs. **A** MCP-1 production in SFMCs cultured for 48 h untreated (UT) or treated with pembrolizumab (Pembro), pembrolizumab + adalimumab (Ada), pembrolizumab + tocilizumab (Toci) (*n* = 10, 2 RA patients, 4 PsA patients, and 4 SpA patients). **A** MCP-1 production in SFMCs cultured for 48 h untreated (UT) or treated with DMSO, pembrolizumab (Pembro), pembrolizumab + tofacitinib (Tofa), or pembrolizumab + baricitinib (Bari) (*n* = 7, 3 PsA patients and 4 SpA patients). **C**, **D** Data were normalized to untreated cultures and expressed as ratios. Data is expressed as median with interquartile range.. Log-transformed ratios were analyzed with the paired Student’s *t*-test. *P-value < 0.05, **P-value < 0.01, ***P-value < 0.001. DMSO, dimethyl sulfoxide; Pembro, pembrolizumab; Ada, adalimumab; Toci, tocilizumab; Tofa, tofacitinib; Bari, baricitinib

We furthermore investigated the cytokine profile by culturing the pembrolizumab treated cells with different cytokine targeting DMARDs used in the treatment of immune-mediated inflammatory arthritis. Adalimumab, tofacitinib, and baricitinib decreased the MCP-1 production (*P* = 0.0004, *P* = 0.033, and *P* = 0.024, respectively). In contrast, in cultures treated with tocilizumab no decrease in MCP-1 production was seen (*P* = 0.7488) (Fig. 6).

## Discussion

**Fig. 7:**
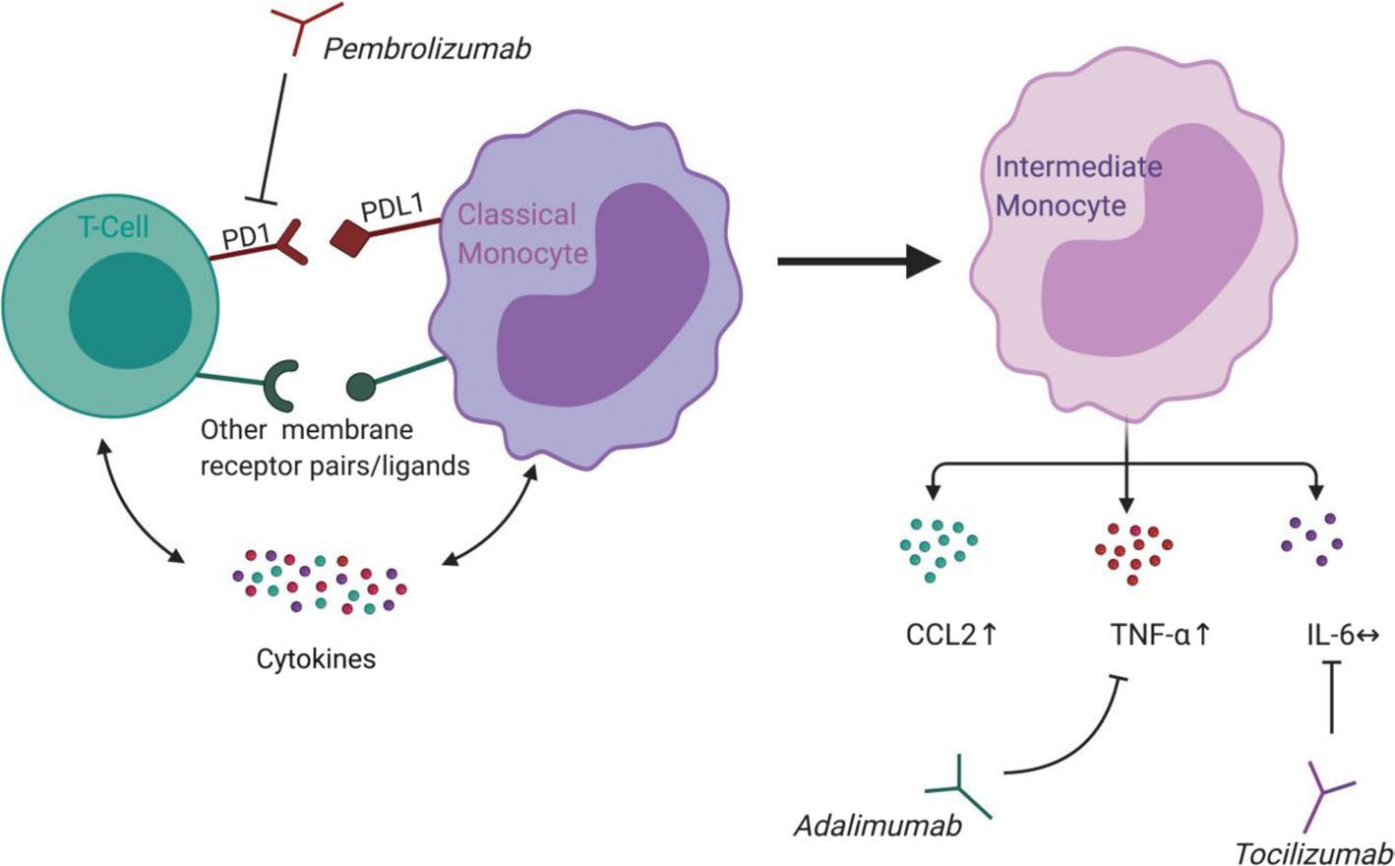
The main findings. Pembrolizumab inhibits the engagement of PD-1 with PD-L1. Removing this immunological brake in SFMC cultures resulted in differentiation of monocytes into intermediate monocytes. The intermediate monocytes showed an increased production of CCL-2 (MCP-1) and TNFα upon pembrolizumab stimulation. No increase was seen in IL-6 production. Inhibiting TNFα production with adalimumab decreased pembrolizumab-induced MCP-1 production. Blocking IL-6 secretion with tocilizumab did not decrease the pembrolizumab-induced immune reactions. Illustration made with BioRendor

Patients with pre-existing inflammatory arthritis often experience disease flare during ICI treatment. This can lead to discontinuation of otherwise well indicated immunotherapy. Identification of the mechanisms of ICI induced arthritis disease flare could guide therapeutic management. We investigated the effect of the PD1 inhibitor pembrolizumab on synovial cells from patients with inflammatory arthritis (Fig. 7).

First, we used SFMCs and PBMCs from patients with inflammatory arthritis and healthy controls to investigate the response to anti-PD-1 treatment with pembrolizumab in vitro. Here, only the SFMCs produced MCP-1 following pembrolizumab treatment. This finding is in line with previous studies showing that pembrolizumab requires an activated immune response to unleash additional immune activation and that the expression of PD-1 and PD-L1 is increased in inflammatory arthritis [6, 29]. We speculate that the difference between response to pembrolizumab in SFMCs and PBMCs is due to upregulated PD1 and PD1 ligands in SFMCs. However, we did not measure PD1 and PDL1 expression on SFMCs used in this study. Therefore, it is not possible to make associations between cellular expression of PD1 and the effect of pembrolizumab. Not all donors showed a response to the treatment. This study was too small to make comparisons of pembrolizumab responses in patients with RA, PsA, and peripheral SpA. However, there were no visual differences in response pembrolizumab in the SFMC cultures from patients with the three disease groups. This study was also too small to make comparisons of pembrolizumab responses in patients treated with a TNF inhibitor at the time of sample collection compared with patients not treated with a TNF inhibitor. The patients included were also not entirely representative of patients seen in the everyday rheumatologic clinic. This is likely because patients in this study were included based on having a large swollen joint. Patients with recurrent inflammation of the knee or ankle will be different compared with patients having primarily symmetric small joint polyarthritis or primarily axial involvement.

Inflammatory arthritis is often associated with joint destruction. Therefore, the role of pembrolizumab was also studied using FLS-PBMC co-cultures and SFMC 21-day cultures. Pembrolizumab did not induce MMP-3 or TRAP production. This implies that pembrolizumab does not activate fibroblasts or osteoclasts and might not lead to a joint-destructive phenotype of arthritis. Nevertheless, joint destruction and fractures have been reported as a result of ICI treatment and activation of osteoclasts in vivo cannot be excluded [30–32]. To our knowledge, there are still no systematic reports on radiographic outcomes in patients with ICI-induced inflammatory arthritis.

Next, we aimed to characterize the cells responsible for MCP-1 production using flow cytometry. We focused on monocytes because these cells have been shown previously to produce MCP-1 [24]. MCP-1 production increased in monocytes following treatment with pembrolizumab, confirming the ELISA results. Furthermore, we identified the intermediate monocytes to be the main producers of MCP-1. Other studies have previously reported that intermediate monocytes express higher levels of both PD-1 and PD-L1 compared to classical monocytes [33]. This may explain why these cells are more responsive to pembrolizumab treatment. As a control, LPS stimulated both subsets of monocytes. This is consistent with expression of Toll like receptor 4 and CD14 in both classical and intermediate monocytes [34]. It is also interesting that pembrolizumab increased the frequency of intermediate monocytes. In RA, intermediate monocytes are expanded in peripheral blood and in synovial fluid, and the frequency of intermediate monocytes in the periphery is associated with disease severity [35, 36]. Further, intermediate monocytes have been shown to be the predominant subset differentiating into inflammatory macrophages in the arthritic joint [37]. However, the function of the different monocyte subsets is still under investigation [38]. The mechanism for the increased production of MCP-1 by monocytes cannot be concluded based on the ex vivo model used in this study. Importantly, we did not explore changes in B cells, T cells, NK cells, or any other cell type. Likely, PD1 inhibition with pembrolizumab exerts effects on several cell subsets leading to upregulation of MCP-1 by intermediate monocytes.

To further clarify the immune response initiated by pembrolizumab, the production of a panel of pro-inflammatory cytokines was evaluated using a multiplex assay. Interestingly, we saw an increase in TNFα production but not in IL-6 production. It is obviously not possible to separate the contribution from monocytes from the contribution from other cells types such as B cells, T cells, and NK cells in the cell culture when measuring supernatants. However, this finding is interesting because previous studies have shown that intermediate monocytes primarily produce TNFα while classical monocytes primarily produce IL-6 [39, 40]. Our findings could thus be explained by activation of intermediate monocytes and increased expression of effector cytokines from these cells. To validate these findings, we tested whether commercially available cytokine inhibitors affected the pembrolizumab-induced induction of MCP-1. Therefore, pembrolizumab treatment was combined with adalimumab (TNFα inhibitor) or tocilizumab (IL-6R inhibitor). TNFα inhibition decreased the MCP-1 production, whereas IL-6R inhibition did not. These findings are in line with clinical studies showing that ICI-induced arthritis can be treated successfully with drugs blocking TNFα [41–43]. Taken together, these observations indicate that TNFα is important for inflammation induced by PD-1 blockade. Our findings imply that IL-6 does not play a significant role in pembrolizumab-induced inflammation. However, clinical studies have shown that inhibition of IL-6 signaling can manage severe immune-related adverse events such as pneumonitis, serum sickness, and cerebritis [44]. A small case series of three patients also reported clinical benefit of tocilizumab treatment in ICI-induced inflammatory arthritis [43]. In our study, the pembrolizumab-induced immune reactions were also dampened by the two JAK inhibitors tofacitinib (JAK1/3 inhibitor) and baricitinib (JAK1/2 inhibitor). It is not possible to identify the cytokines responsible for the effect of JAK inhibition in this study. However, JAK signaling is utilized by several of the cytokines measured in the V-plex assay including IL-10, IL-12p70, IFN-γ, IL-13, IL-2 and IL-4, and IL-6. Therefore, JAK inhibition could prevent MCP-1 secretion in the SFMC cultures by preventing signaling through these cytokine receptors. It is not known whether the mechanisms demonstrated in these short duration models truly reflect treatment effects in vivo where IRAEs and response to DMARDs often occur after several weeks. Specifically, the sample size was too small to study differences in TNFα and IL-6 secretion between RA, PsA and peripheral SpA. Randomized trials are obviously needed to elucidate the efficacy of different DMARDs in these diseases.

Whether DMARDs and other immunomodulatory agents affect the anti-cancer potential of checkpoint inhibitors is still not understood. Recently, MCP-1 and TNFα levels have been associated with the overall survival following treatment with ICIs [45]. This could imply that an inhibition of the monocytes with DMARDs may interfere with the mode of action of ICI treatment. On the other hand, the increased levels of MCP-1 and TNFα could also reflect a general bystander immune activation initiated by the cancer treatment. This would imply that treating the IRAEs would not interfere with ICI anti-cancer effects.

## Conclusions

Taken together, we found that intermediate monocytes are activated when SFMC cultures are treated with pembrolizumab. Pembrolizumab-induced the production of several cytokines and TNFα seems to be more important than IL-6. Our findings indicate that flares in patients with pre-existing inflammatory arthritis involve monocyte activation and could be managed with TNFα neutralization. MMP3 production from FLSs and TRAP activity in osteoclasts were not increased by pembrolizumab suggesting that joint destructive processes might not be induced by PD1 inhibition. This was a relatively small study and needs replication in larger studies and in arthritis in vivo models.

## Supplementary Information

**Additional file 1: Figure S1**. MCP-1 production in SFMCs (*n*=16, 3 SpA patients, 5 PsA patients, and 8 RA patients) following stimulation with pembrolizumab. Data were normalized to untreated cultures and expressed as ratios. Data is presented as median with interquartile range. RA, Rheumatoid Arthritis; SpA, Spondyloarthritis; PsA, Psoriatic Arthritis. **Figure S2**. Representative CD14 vs. CD16 dot plot of monocyte subsets in PBMCs. Monocytes were gated as CD45+/live/TLR-2+/singlets. **Figure S3**. Gating strategy. Monocytes were characterized as CD45+/live/TLR-2+/singlets. **Figure S4**. (A) Representative MCP-1 vs SSC-A dotplot of MCP-1 production in all monocytes in each culture. (B) Frequency of MCP-1 + cells in all monocytes. (C) Data were normalized to untreated cultures and expressed as ratios. All data are expressed as median with interquartile range. * *P*-value < 0.05, ** *P*-value < 0.01, *** *P*-value < 0.001. UT, untreated; Pembro, Pembrolizumab.

## Data Availability

Please contact corresponding author for data requests.

## Abbreviations

Ada: Adalimumab
Bari: Baricitinib
BSA: Bovine serum albumin
CM: Classical monocytes
CRP: C-reactive protein
CTLA-4: Cytotoxic T lymphocyte-associated antigen 4
DAS28-CRP: Disease Activity Score 28 – C-reactive protein
DMARDs: Disease-modifying anti-rheumatic drugs
DMEM: Dulbecco’s Modified Eagle Medium
DMSO: Dimethyl sulfoxide
ELISA: Enzyme-linked immunosorbent assay
FCS: Fetal calf serum
FLS: Fibroblast-like synoviocytes
ICI: Immune checkpoint inhibitors
IFN: Interferon
IgG: Immunoglobulin G
IL: Interleukin
IL-6R: Interleukin-6 receptor
IM: Intermediate monocytes
IMIDs: Immune-mediated inflammatory diseases
IQR: Interquartile range
IRAEs: Immune-related adverse events
JAK: Janus-activated kinase
LPS: Lipopolysaccharide
M-CSF: Macrophage colony-stimulating factor
MCP-1: Monocyte chemoattractant-protein 1
MMP3: Matrix metalloproteinase 3
PBMCs: Peripheral blood mononuclear cells
PD-1: Programmed cell death protein 1
PD-L1: Programmed cell death ligand 1
PD-L2: Programmed cell death ligand 2
Pembro: Pembrolizumab
PsA: Psoriatic arthritis
RA: Rheumatoid arthritis
RANKL: Receptor activator of nuclear factor κ-B ligand
RT: Room temperature
SFMCs: Synovial fluid mononuclear cells
SpA: Spondyloarthritis
TLR: Toll-like receptor
TNF: Tumor necrosis factor
Toci: Tocilizumab
Tofa: Tofacitinib
TRAP: Tartrate-resistant acid phosphatase
UT: Untreated
