# Supplementary material for: Tumor necrosis factor alpha neutralization attenuates immune checkpoint inhibitor-induced activation of intermediate monocytes in synovial fluid mononuclear cells from patients with inflammatory arthritis
Source: Arthritis Res Ther. 2022 Feb 14;24:43. doi: 10.1186/s13075-022-02737-6 (PMC8842914; doi:10.1186/s13075-022-02737-6)
Supplement: Supplementary file 1 — Additional file 1: Figure S1. MCP-1 production in SFMCs (n=16, 3 SpA patients, 5 PsA patients, and 8 RA patients) following stimulation with pembrolizumab. Data were normalized to untreated cultures and expressed as ratios. Data is presented as median with interquartile range. RA, Rheumatoid Arthritis; SpA, Spondyloarthritis; PsA, Psoriatic Arthritis. Figure S2. Representative CD14 vs. CD16 dot plot of monocyte subsets in PBMCs. Monocytes were gated as CD45+/live/TLR-2+/singlets. Figure S3. Gating strategy. Monocytes were characterized as CD45+/live/TLR-2+/singlets. Figure S4. (A) Representative MCP-1 vs SSC-A dotplot of MCP-1 production in all monocytes in each culture. (B) Frequency of MCP-1 + cells in all monocytes. (C) Data were normalized to untreated cultures and expressed as ratios. All data are expressed as median with interquartile range. * P-value < 0.05, ** P-value < 0.01, *** P-value < 0.001. UT, untreated; Pembro, Pembrolizumab. [file 13075_2022_2737_MOESM1_ESM.docx]

**Supplementary figures and legends**

***Figure S1: MCP-1 production in SFMCs (n=16, 3 SpA patients, 5 PsA patients, and 8 RA patients) following stimulation with pembrolizumab.*** *Data were normalized to untreated cultures and expressed as ratios. Data is presented as median with interquartile range. RA, Rheumatoid Arthritis; SpA, Spondyloarthritis; PsA, Psoriatic Arthritis.*

***Figure S2:*** *Representative CD14 vs. CD16 dot plot of monocyte subsets in PBMCs. Monocytes were gated as CD45+/live/TLR-2+/singlets.*

**

***Figure*** ***S3****: Gating strategy. Monocytes were characterized as CD45+/live/TLR-2+/singlets.*

**

***Figure S4: (A)*** *Representative MCP-1 vs SSC-A dotplot of MCP-1 production in all monocytes in each culture.* ***(B)*** *Frequency of MCP-1 + cells in all monocytes.* ***(C)*** *Data were normalized to untreated cultures and expressed as ratios. All data are expressed as median with interquartile range. * p-value < 0.05, ** p-value < 0.01, *** p-value < 0.001. UT, untreated; Pembro, Pembrolizumab.*
